# The synergistic interaction between ACE and TMPRSS2 polymorphisms increases the risk of severe COVID-19

**DOI:** 10.1371/journal.pone.0343590

**Published:** 2026-02-24

**Authors:** Odonchimeg Bayaraa, Chimedlkhamsuren Ganbold, Bayarlakh Byambadorj, Zolzaya Battulga, Ichinnorov Dashtseren, Sarantuya Jav

**Affiliations:** 1 Department of Pulmonology and Allergy, School of Medicine, Mongolian National University of Medical Science, Ulaanbaatar, Mongolia; 2 The Center of Pulmonology and Allergy, First Central Hospital of Mongolia, Ulaanbaatar, Mongolia; 3 Department of Molecular Biology and Genetics, School of Biomedicine, Mongolian National University of Medical Science, Ulaanbaatar, Mongolia; 4 Institute of Biomedical Sciences, Mongolian National University of Medical Science, Ulaanbaatar, Mongolia; Universidade Federal do Para, BRAZIL

## Abstract

Within a few years after the pandemic outbreak, several of evidence have found to suggest that the genetic factors influence the severity and mortality rate of COVID-19. In particular, the identification of genetic markers that increase the risk of severe or critical COVID-19 is important for public health management during the pandemic. By August 2021, 88.9% of Mongolian population had been vaccinated. Therefore, we conducted this study to compare the polymorphisms of candidate genes by selecting people who developed mild or severe COVID-19 within this vaccinated population. A total of 90 patients with severe COVID-19, 95 patients with mild COVID-19, and 90 asymptomatic patients were participated in present cross-sectional study. rs4646994, rs4240157, rs41423247, rs56149945, rs10052957, rs12329760, rs4303795, rs75603675 and rs17854725 polymorphisms of the *ACE*, *ACE2*, *NR3C1* and *TMPRSS2* genes were genotyped. Genotyping performed by real-time PCR, RFLP and allele-specific PCR methods. Odds ratio, 95% confidence interval, p value were calculated using logistic regression analysis. SNP-SNP interaction were explored using multi-dimensional reduction analysis. P values for multivariate model was corrected by Bonferroni correction. Totally 10 polymorphisms of above-mentioned genes were genotyped among the groups. Only A/C genotype frequencies of rs75603675 were significantly different between groups. Compared with mild COVID-19 group, the participants who carrying A/C genotype of rs75603675 had 3.58-fold (95% CI, 1.38–9.29, p = 0.009) higher risk for severe COVID-19. In addition, the synergistic interaction (RERI = 2.573; AP = 0.934; S = 8.352) was observed between D/D or I/D genotype of rs4646994 and A/C genotype of rs75603675, which combination (OR=4.88, 95% CI, 1.38–18.01, p = 0.014, Power = 91.1%) was associated with increased risk of severe COVID-19 after Bonferroni correction. Our result suggesting that the combination of rs4646994 of the *ACE* gene and rs75603675 of the *TMPRSS2* gene is associated with increased risk of severe COVID-19.

## Introduction

The coronavirus disease 2019 (COVID-19) is caused by infection with the severe acute respiratory syndrome coronavirus 2 (SARS-CoV-2) and according to WHO data, as of 15 September 2024, approximately 776 million cases and 7.07 million deaths had been recorded worldwide [1]. Since the first case confirmed on March 10, 2020, more than 1 million cases of COVID-19 infection have been recorded in Mongolia until now [1,2]. COVID-19 appears notable inter-individual variability in clinical manifestations, ranging from asymptomatic diseases to life-threatening condition and death, with more severe courses being associated with age, male sex, and comorbidities. Besides these risk factors, intrinsic characteristics of the virus as well as genetic factors of the host are expected to account for COVID-19 clinical heterogeneity [3].

Higher expression, polymorphisms, mutations, and deletions of several genes are linked with the susceptibility, severity, and clinical outcomes of COVID-19 [4]. Severe COVID-19 have been related with a varied expression of several genes and their alleles such as HLA, Angiotensin-converting enzyme-2 (ACE-2), cellular proteases, and immune response proteins [4]. Elevated expression of ACE2 and TMPRSS2, as well as the increase of pro-inflammatory cytokines might indicate the progression of COVID-19 into a severe and critical stage [5]. A thorough understanding of the role play between different genetic factors and the progression of COVID-19 infection is crucial to develop new therapeutic options, strategies for disease prevention, and diagnostic, thereby starting medical intervention during the early course of the disease, ultimately leading to better clinical outcomes [4]. Quite of few evidences have found to suggest that the host genetic factors influence the severity and mortality rate of COVID-19. A number of the polymorphisms of candidate genes such as *ACE*, *ACE2* and *TMPRSS2*, were identified as genetic factor for the susceptibility to COVID-19 and its severity [4,6].

Angiotensin-converting enzyme-2 (ACE2) receptor is a key protein involved in SARS-CoV-2 entry into host cells. This receptor is encoded *ACE2* gene and the polymorphisms affecting the receptor expression and binding affinity to target proteins, might have influence on the pathogenesis of COVID-19 [7]. Recent studies reported rs4240157 polymorphism was an associated with increased severity of COVID-19 [8,9]. In addition, angiotensin-converting enzyme (ACE) increases the production of angiotensin-II (Ang-II), which leads to promote inflammatory process and downregulation of ACE2. Upregulated ACE expression may associated with increased the risk of severe COVID-19 disease [10]. The rs4646994 polymorphism has been commonly studied with COVID-19 and it was reported that the deletion allele of this polymorphism associated with susceptibility to COVID-19 and mortality [11].

*TMPRSS2* gene encodes transmembrane protease serine 2 (TMPRSS2), which is play an important role for *Influenza* virus, HCoV-229E, MERS-CoV, SARS-CoV and SARS-CoV-2 to enter host cells. TMPRSS2 cleaves the Spike protein of SARS-CoV-2 and facilitates its entry into host cells [12]. Number of polymorphisms that located on *TMPRSS2* gene, were explored with COVID-19 and significant associations were found, especially for rs12329760 and rs75603675 [13]. In this study, we investigated the combined effects of polymorphisms of *ACE*, *ACE2, NR3C1* and *TMPRSS2* genes in association with the severity of COVID-19 disease and aimed to identify significant combinations.

## Materials and methods

### Study subject

This cross-sectional study was conducted following the STREGA (STrengthening the REporting of Genetic Association studies) guidelines to ensure standardized reporting and transparency. Between 1^st^ of June and 31^st^ of December 2021, a total of two hundred seventy five people infected with SARS-CoV-2 were collected using non-probability sampling in this study. The total sample consisted of 90 patients with severe COVID-19 and 95 patients with mild COVID-19 who were hospitalized at the First State Central Hospital of Mongolia, and 90 asymptomic volunteers who were visited as outpatient clinic. Inclusion criteria for the study were (1) patients infected with delta variant of SARS-CoV-2 which was confirmed by RT-qPCR test, (2) the course of the disease corresponds to one of the following groups according to the living guidance for clinical management of COVID-19 issued by WHO and the COVID-19 diagnosis and treatment guidelines approved by the Minister of Health (Order number: A/549). The severe COVID-19 group included patients with oxygen saturation in room air is less than 90%, signs of pneumonia and severe respiratory distress confirmed by physical examination and X-ray. In mild COVID-19 group, the patients without signs of severe or critical disease participated. The asymptomatic group included people infected with SARS-CoV-2 who exhibited no symptoms. Data related to the participants’ COVID-19 vaccination status was obtained from the National Immunization Registry electronic database, Mongolia. Pregnant women and those who had not been vaccinated against SARS-CoV-2 were excluded from the study. Also, individuals who immunocompromised (including those on an immunosuppressive therapy), patients with malignancy of any kind or had HIV were not participated in this study. All participants voluntarily agreed to participate in the study and signed an informed consent form. This study was approved by the Research Ethics Committee of Mongolian National University of Medical Science (Protocol number: 2022/3-06) and Medical Ethics Committee of The Ministry of Health (Protocol number: 23/002) in accordance with the Declaration of Helsinki.

### Genotyping of the variants

Peripheral whole blood samples were collected in EDTA tube. Nucleic acid was extracted using AccuPrep® Genomic DNA Extraction Kit (Cat. No. K-3032G, Bioneer Corporation) according to the manufacturer`s manual protocol. A total of 10 polymorphisms located in four genes were genotyped using PCR, RFLP and qPCR methods. PCR and qPCR reactions were carried out using AccuPower® PCR Master Mix (Cat. No. K-2018, Bioneer Corporation) and AccuPower® Plus DualStar™ qPCR Master Mix (Cat. No. K-6608, Bioneer Corporation). In addition, Bioneer Corporation synthesized all primers and probes by the order. The products were analyzed by electrophoresis with an agarose gel (Cat. No. C-9100-1, Bioneer) and visualized with ethidium-bromide staining. We used PCR, which were previously described by Mir MM et al, to determine the genotypes of rs4646994 of *ACE* and rs4240157 of *ACE2* [9]. For rs56149945 and rs41423247 of *NR3C1* gene, RFLP analysis were performed [14]. The specific fragments were amplified by PCR using the primers, and then the amplicons were digested by the restriction enzyme. Digested or amplified DNA products were analyzed by gel electrophoresis and genotypes were discriminated by the amplicon`s length. All primers, probes and restriction enzymes that were used for genotyping, summarized in S1 Table. The rs10052957 and rs6189/6190 polymorphisms of *NR3C1* gene, were explored using qPCR with TaqMan probes [15]. Allele or genotypic discrimination of rs17854725, rs75603675, rs12329760 and rs4303795 polymorphisms of *TMPRSS2* were performed using amplification refractory mutation system-PCR and RFLP [16].

### Statistical analysis

STATA 13.0 (StataCorp, USA) and Microsoft Excel (Microsoft Corporation, USA) software were used for statistical analysis. One-way ANOVA test was performed to compare numerical data such as age, body mass index (BMI) between groups. Hardy-Weinberg Equilibrium (HWE) was calculated using the direct count of allele and genotype frequency and standard parameters of the SNPAlyze 9.0 (Dynacom, Japan) software. Comparison of allelic and genotypic frequency between groups were performed by Pearson`s Chi-square test (x2) and the Fisher’s exact test. The alpha level was set at 0.05, and statistical significance was considered when P value was less than 0.05. Odds ratios (OR) and 95% confidence interval (CI) were calculated by a logistic regression. For the multivariate model, adjusted odds ratios (ORs) and 95% confidence intervals (CIs) were calculated, using for age, gender, BMI, education level, occupation type, vaccination status, the interval between vaccine doses, vaccine type across all doses, smoking status, and alcohol use as adjustment variables. Bonferroni correction was applied to correction of the p-values. A method-four model strategy that described by Nobuyuki Horita was used to select the genetic model [17]. Multifactor dimensionality reduction (MDR) analysis was performed using MDR 3.0.2 software to identify the SNP–SNP combined effect on the COVID-19 risk. To minimize the risk of false positives, data were generated using a 10-fold cross-validation procedure. The best model was selected based on maximum cross-validation consistency (CVC), training balance accuracy (TrBA) and testing balance accuracy (TeBA). The statistical power was calculated using a post-hoc test to estimate the strength of the associations. Potential additive interactions between SNPs in relation to COVID-19 risk were evaluated using the relative excess risk due to interaction (RERI), the synergy index (S), and the proportion attributable to interaction (AP), according to the formulas described by Linda Kalilani [18].

## Results

Ninety-patients with severe COVID-19, ninety-five patients with mild COVID-19 and ninety-patients with asymptomatic volunteers participated in this study. The general data of the study participants are compared between the groups and shown in Table 1. The study groups were similar in terms of age, gender ratio, BMI categories, education level, smoking and alcohol consumption. In addition, number of participants with the comorbidities such as coronary heart disease, renal, liver and pulmonary chronic diseases, were compared between groups and no significant differences has found. Arterial hypertension was most common comorbidity and it was significantly different between severe and mild COVID-19 groups (OR: 1.83; 95% CI: 1.02–3.29; p = 0.043). Type II diabetes had higher prevalence among severe COVID-19 patients compared with mild COVID-19.

**Table 1:** General characteristics of the groups.

Characteristics	Severe COVID 19 (n = 90)	Mild COVID 19 (n = 95)	Asymptomatic (n = 90)	P value
Age (years)	56.72 ± 11.06	57.56 ± 11.55	54.26 ± 15.56	0.197^a^
Gender				
Male	39 (43.3)	35 (36.8)	37 (41.1)	0.657^c^
Female	51 (56.7)	60 (63.2)	53 (58.9)	
Body mass index categories				
Underweight	1 (1.11)	1 (1.05)	4 (4.45)	0.318^d^
Healthy weight	21 (23.33)	22 (23.16)	30 (33.33)	
Overweight	33 (36.67)	35 (36.84)	28 (31.11)	
Obese	35 (38.89)	37 (38.95)	28 (31.11)	
Education				
Primary education	0 (0.0)	1 (0.01)	3 (0.03)	0.269^d^
Secondary education or college/senior	47 (52.2)	48 (50.5)	53 (58.9)	
Tertiary education	43 (47.8)	46 (49.4)	34 (37.8)	
Occupational type				
Physical	38 (42.2)	39 (41.1)	42 (46.7)	0.721^c^
Not-physical	52 (57.8)	56 (48.9)	48 (53.3)	
Comorbidities				
Coronary heart disease	5 (5.56)	6 (6.32)	5 (5.56)	0.968^d^
Arterial hypertension	56 (62.22)	45 (47.37)	47 (52.22)	0.12^c^
Type I diabetes	3 (3.33)	2 (2.11)	1 (1.11)	0.593^d^
Type II diabetes	19 (21.11)	13 (13.68)	9 (10.0)	0.103^c^
Chronic obstructive pulmonary disease	19 (21.11)	16 (16.84)	20 (22.22)	0.625^c^
Asthma	15 (16.67)	14 (14.74)	12 (13.33)	0.819^c^
Cerebrovascular disease	4 (4.44)	2 (2.11)	2 (2.22)	0.572^d^
Chronic renal disease	3 (3.33)	8 (8.42)	6 (6.67)	0.347^d^
Chronic liver disease	11 (12.22)	10 (10.53)	5 (5.56)	0.282^d^
Current smoker				
Yes	16 (17.8)	17 (17.9)	25 (27.8)	0.166^c^
No	74 (82.2)	78 (92.1)	65 (72.2)	
Alcohol abuse				
Yes	80 (88.9)	88 (92.6)	81 (90.0)	0.669^c^
No	10 (11.1)	7 (7.4)	9 (10.0)	

The values were given as numbers (proportion) or mean ± standard deviation.

^a^ P value was calculated by One-way ANOVA test.

^b^ P value was calculated by Kruskal-Wallis test.

^c^ P value was calculated by Chi square (x2) test.

^d^ P value was calculated by Fisher`s exact test.

All study participants were previously vaccinated. The data of vaccination status and comparison between groups were shown in Table 2. The participants were categorized based on whether they had received one, two, or three doses of the vaccine, and the comparison among the three groups showed no significant difference (p = 0.646). No significant difference was observed in the interval between vaccine doses when measured in days (p > 0.05). However, when participants were categorized by vaccine type, a significant difference was found in the first and second doses. Specifically, the number of individuals who had received inactivated (Sinopharm) vaccines for the first and second doses was relatively lower, whereas those who had received viral vector (AstraZeneca) vaccines were slightly more numerous in the mild COVID-19 group (p = 0.002). Therefore, to minimize the potential confounding effects of COVID-19 vaccine-related variables on the association between COVID-19 severity and genetic alleles or genotypes, we applied multivariate analysis and Bonferroni correction in further comparisons. Also, the frequencies of clinical manifestations in the severe and mild COVID-19 groups are presented in S2 Table.

**Table 2:** Vaccination status of the groups.

Characteristics	Severe COVID 19 (n = 90)	Mild COVID 19 (n = 95)	Asymptomatic (n = 90)	P value
One vaccine dose	2 (2.22)	0 (0.0)	2 (2.22)	0.646^d^
Two vaccine doses	29 (32.22)	34 (35.79)	28 (31.11)	
Three vaccine doses	59 (65.56)	61 (64.21)	60 (66.67)	
Interval between 1^st^ and 2^nd^ dose (days)	41.82 ± 69.04	39.66 ± 49.69	30.23 ± 9.77	0.062^b^
Interval between 2^nd^ and 3^rd^ dose (days)	185.17 ± 85.83	164.03 ± 55.09	157.53 ± 44.49	0.115^b^
Vaccine type				
First dose				
Inactivated (Sinopharm)	72 (80.0)	56 (58.94)	71 (78.89)	0.002^d^
Viral vector (AstraZeneca)	5 (5.56)	26 (27.37)	11 (12.22)	
Viral vector (Sputnik V)	2 (2.22)	2 (2.11)	2 (2.22)	
mRNA (Pfizer-BioNTech)	11 (12.22)	11 (11.58)	6 (6.67)	
Second dose				
Inactivated (Sinopharm)	69 (76.67)	54 (56.84)	69 (76.67)	0.002^d^
Viral vector (AstraZeneca)	5 (5.56)	26 (27.37)	11 (12.22)	
Viral vector (Sputnik V)	2 (2.22)	2 (2.11)	2 (2.22)	
mRNA (Pfizer-BioNTech)	12 (13.33)	13 (13.68)	6 (6.67)	
Third dose				
Inactivated (Sinopharm)	5 (5.56)	5 (5.26)	4 (4.44)	0.142^d^
Viral vector (AstraZeneca)	1 (1.11)	9 (9.47)	5 (5.56)	
mRNA (Pfizer-BioNTech)	53 (58.89)	47 (49.47)	51 (56.67)	

The values were given as numbers (proportion) or mean ± standard deviation.

^b^ P value was calculated by Kruskal-Wallis test.

^d^ P value was calculated by Fisher`s exact test.

The frequencies of alleles and genotypes of all 10 polymorphisms of *ACE*, *ACE2*, *NR3C1*, and *TMPRSS2* genes were compared between three groups. No statistically significant differences were observed when comparing allele frequencies between groups, and the comparative results are shown in Table 3.

**Table 3:** Allelic frequencies of SNPs among groups.

Gene	refSNP ID	Allele	Frequency			OR (95% CI)^a^				HWE p value
			S (n = 180)	M (n = 190)	A (n = 180)	S vs M	S vs A	M vs A	S + M vs A	
ACE	rs4646994	D	0.35	0.326	0.289	1.11 (0.72–1.71)	1.33 (0.85–2.07)	1.19 (0.77–1.86)	1.26 (0.85–1.85)	0.36
ACE2	rs4240157	C	0.394	0.373	0.344	1.09 (0.72–1.66)	1.24 (0.81–1.90)	1.13 (0.74–1.74)	1.18 (0.82–1.72)	0.289
NR3C1	rs41423247	C	0.306	0.274	0.283	1.16 (0.74–1.83)	1.11 (0.71–1.75)	0.95 (0.60–1.50)	1.03 (0.69–1.53)	0.205
NR3C1	rs56149945	G	0.017	0.005	0.011	3.20 (0.33–31.08)	1.51 (0.25–9.14)	0.47 (0.04–5.24)	0.97 (0.17–5.36)	0.854
NR3C1	rs10052957	A	0.111	0.089	0.094	1.27 (0.64–2.51)	1.19 (0.61–2.37)	0.94 (0.47–1.91)	1.06 (0.58–1.95)	0.225
TMPRSS2	rs12329760	A	0.478	0.421	0.478	1.25 (0.83–1.89)	1.00 (0.66–1.51)	0.79 (0.53–1.19)	0.88 (0.62–1.27)	0.299
TMPRSS2	rs4303795	G	0.139	0.111	0.106	1.27 (0.69–2.41)	1.37 (0.72–2.58)	1.05 (0.55–2.03)	1.20 (0.68–2.12)	0.626
TMPRSS2	rs75603675	A	0.528	0.489	0.483	1.17 (0.78–1.75)	1.19 (0.79–1.81)	1.02 (0.68–1.54)	1.10 (0.77–1.58)	0.116
TMPRSS2	rs17854725	A	0.811	0.784	0.744	1.18 (0.71–1.96)	1.47 (0.89–2.43)	1.24 (0.77–2.02)	1.35 (0.89–2.06)	0.094

S, Severe COVID-19; M, Mild COVID-19; A, Asymptomatic; OR, Odd`s ratio; 95% CI, 95% confidence interval.

^a^ Odd’s ratio and confidence interval was calculated by logistic regression.

For indicating the significant difference, p value noted as *p < 0.05, **p < 0.01 or ***p < 0.001.

However, comparing the genotype frequency between groups, the A/C genotype of *TMPRSS2* gene rs75603675 polymorphism was significantly different. The A/C genotype of the rs75603675 polymorphism had a relatively high frequency in the severe COVID-19 group compared with mild COVID-19 group (0.411 vs 0.242, respectively). Univariate analysis showed that the A/C genotype increases the risk of severe COVID-19 by 2.18 times (OR: 2.18; 95% CI: 1.16–4.1; p = 0.014). In addition, it was observed that A/C genotype of rs75603675 was also significantly different between the groups and increase the risk of severe COVID-19 by 3.58 times (OR: 3.58; 95% CI: 1.38–9.29; p = 0.009) by multivariate analysis. However, no difference was observed between the severe COVID-19 and asymptomatic groups for A/C genotype frequency. The genotype frequencies of other polymorphisms were similar between the groups, and no statistically significant differences were observed in either the univariate or multivariate analyses. Genotype frequencies and comparative results are shown in Table 4 and S3 Table.

**Table 4:** Comparison of genotype frequencies of SNPs between groups.

refSNP ID	Model	Genotype	Frequency			S vs M		S vs A	
			S N = 90	M N = 95	A N = 90	OR (95% CI)^a^	aOR (95% CI)^b^	OR (95% CI)^a^	aOR (95% CI)^b^
rs4646994	Dominant	D/D + I/D	0.556	0.516	0.444	1.17 (0.66–2.09)	1.25 (0.56–2.78)	1.56 (0.87–2.81)	2.01 (0.87–4.67)
rs4240157	Recessive	C/C	0.078	0.032	0.067	2.59 (0.65–10.33)	2.20 (0.37–13.04)	1.18 (0.38–3.66)	1.09 (0.23–5.13)
rs41423247	Recessive	C/C	0.122	0.095	0.078	1.33 (0.52–3.38)	0.60 (0.16–2.34)	1.65 (0.61–4.47)	1.49 (0.28–7.82)
rs56149945	Overdominant	A/G	0.033	0.011	0.022	3.24 (0.33–31.75)	2.22 (0.16–29.9)	1.52 (0.25–9.30)	1.17 (0.06–22.3)
rs10052957	Recessive	A/A	0.033	0.021	0.011	1.60 (0.26–9.83)	1.08 (0.06–19.3)	3.07 (0.31–30.1)	1.20 (0.07–20.8)
rs6189/6190	Recessive	Non GG	0.022	0.021	0.011	1.06 (0.15–7.66)	1.64 (0.09–30.92)	2.02 (0.18–22.7)	0.88 (0.04–18.9)
rs12329760	Recessive	A/A	0.267	0.200	0.211	1.26 (0.64–2.49)	1.76 (0.65–4.73)	1.18 (0.59–2.34)	1.25 (0.43–3.66)
rs4303795	Overdominant	A/G	0.278	0.179	0.189	1.76 (0.88–3.55)	1.69 (0.82–3.50)	1.65 (0.82–3.33)	1.63 (0.77–3.47)
rs75603675	Overdominant	A/C	0.411	0.242	0.367	2.18 (1.16–4.10)*	3.58 (1.38–9.29)*	1.21 (0.66–2.19)	1.27 (0.55–2.93)
rs17854725	Dominant	A/A	0.756	0.695	0.667	1.36 (0.71–2.60)	1.12 (0.45–2.79)	1.55 (0.81–2.96)	1.02 (0.40–2.58)

S, Severe COVID-19; M, Mild COVID-19; A, Asymptomatic; OR, Odd`s ratio; aOR, Adjusted Odd`s ratio; 95% CI, 95% confidence interval.

^a^ Odd’s ratio and confidence interval was calculated by logistic regression.

^b^ Calculated by multivariate logistic regression and adjusted for age, gender, BMI, education level, occupational type, vaccination status and interval between vaccine doses, vaccine type of all doses, smoking status and alcohol abuse.

* Significant after Bonferroni correction.

**Fig 1:**
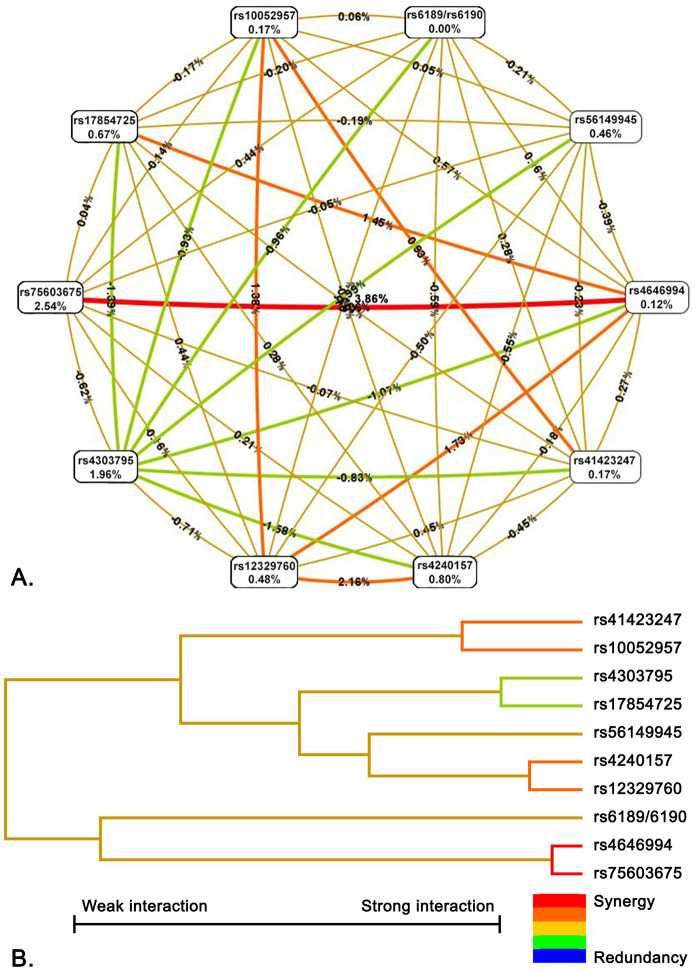
SNP-SNP interaction network and dendrogram of 10 polymorphisms in the comparison between severe and mild COVID-19 groups. **A.** Entropy-based SNP–SNP interaction network: The network depicts interactions among 10 polymorphisms of the studied genes in the comparison between severe and mild COVID-19 groups. The graph shows the percentage of entropy for both independent effects and their interactions, where a positive entropy percentage indicates synergistic interaction, and a negative percentage indicates redundancy. The best predictive model was composed of rs75603675 and rs4646994. In the graph, red and orange colors denote synergistic interactions, reflecting evidence of epistasis. The gold color represents a neutral or midpoint interaction, green indicates moderate redundancy, and blue denotes the highest level of redundancy. **B.** Dendrogram of SNP–SNP interactions: The dendrogram displays a color gradient illustrating the continuum from synergy to redundancy: red represents a high degree of synergy (greater than additive effect), whereas green indicates redundancy (less than additive effect). Strongly interacting SNP pairs, such as rs4646994 and rs75603675, are positioned close together at the leaves of the tree, while weakly interacting SNPs appear farther apart.

Further, we performed MDR analysis comparing interactions between polymorphisms and found interesting results. Fig 1 shows the SNP-SNP interaction network of polymorphisms between severe and mild COVID-19 groups. Highest degree of effect is attributed to rs75603675 of *TMPRSS2* gene by the entropy-based analysis. Only rs75603675 eliminates 2.54% of the total entropy. On the other hand, the rs4646994 polymorphism of the *ACE* gene removes almost zero entropy, but when combined with rs75603675, it removed a total of 6.52% entropy which indicates that we found the strong positive interaction between these two polymorphisms among mild and severe COVID-19 groups.

Using MDR analysis, the best 10-combinatory models among the 10 polymorphisms were identified in comparison between severe and mild COVID-19 groups, and their cumulative odds ratios were calculated. The above results are presented in Table 5. The cumulative odds ratios of these models ranged from 2.18 to 540; however, only the rs75603675 and rs4646994 model demonstrated a significantly synergistic interaction. Also, it was determined that cumulative odd`s ratio of rs75603675 of *TMPRSS2* and rs4646994 of the *ACE* combinatory model, was 3.43 (95% CI: 1.87–6.28; p < 0.001).

**Table 5:** Best models of SNP-SNP interactions between severe and mild COVID-19 groups.

Models	Training Bal.Acc.	Testing Bal.Acc.	OR (95% CI)	Sign test (p)	CVC	Chi-square
rs75603675	0.585	0.585	2.18 (1.16–4.10)	0.014	10/10	6.02
rs75603675 & rs4646994*	0.649	0.633	3.43 (1.87–6.28)	<0.001	10/10	16.46
rs75603675, rs4646994 & rs10052957	0.696	0.579	5.11 (2.72–9.60)	<0.001	7/10	27.21
rs4646994, rs4240157, rs12329760 & rs75603675	0.772	0.569	12.89 (6.04–27.48)	<0.001	5/10	52.55
rs4646994, rs41423247, rs4240157, rs12329760 & rs75603675	0.842	0.534	37.15 (14.47–95.41)	<0.001	8/10	83.43
rs4646994, rs41423247, rs4240157, rs12329760, rs17854725 & rs75603675	0.894	0.519	155.05 (35.19–683.16)	<0.001	5/10	109.34
rs4646994, rs41423247, rs4240157, rs12329760, rs17854725, rs10052957 & rs75603675	0.940	0.586	233.81 (67.88–805.3)	<0.001	10/10	140.39
rs4646994, rs41423247, rs4240157, rs12329760, rs17854725, rs4303795, rs10052957 & rs75603675	0.954	0.539	386.75 (100.49–1488.34)	<0.001	9/10	150.74
rs4646994, rs41423247, rs4240157, rs12329760, rs17854725, rs4303795, rs10052957, rs56149945 & rs75603675	0.958	0.545	521.33 (120.89–2248.07)	<0.001	8/10	154.42
rs4646994, rs41423247, rs4240157, rs12329760, rs17854725, rs4303795, rs10052957, rs56149945, rs6189/6190 & rs75603675	0.958	0.551	540.0 (116.71–2498.46)	<0.001	10/10	140.03

Training Bal. ACC, Training Balanced Accuracy; Testing Bal. ACC, Testing Balanced Accuracy; CV, Cross Validation Consistency.

* The model exhibiting synergistic interaction was identified by MDR analysis, RERI, AP and S index.

Based on previous result, the rs75603675 and rs4646994 model was stratified for each genotype combination, and a multivariate analysis was performed to compare between the severe and mild COVID-19 groups. RERI, AP and S index were showed the presence of greater than additive effect, indicating a synergistic interaction between rs75603675 and rs4646994 (RERI = 2.573; AP = 0.934; S = 8.352). Compared with the non-A/C and I/I genotype combination, the A/C and D/D or I/D combination was observed to be associated with an increased risk of severe COVID-19. By the multivariate analysis, the results revealed that individuals carrying the A/C genotype of rs75603675 and the D/D or I/D genotype of rs4646994 had a 4.88-fold (OR: 4.88; 95% CI: 1.38–18.01; p = 0.014, Power = 91.1%) higher risk of developing severe COVID-19 compared to other genotypic combinations (Table 6). No significant differences were observed for other combinations.

**Table 6:** The cumulative effect of rs75603675 and rs4646994 combination on severe COVID-19 compared with mild COVID-19.

rs75603675	rs4646994	Severe COVID 19 (n = 90)	Mild COVID 19 (n = 95)	OR (95% CI)	p value	aOR (95% CI)	p value	Power
**RERI = 2.573; AP = 0.934; S = 8.352**								
Non A/C	I/I	37 (41.11)	34 (35.79)	1	**–**	1	**–**	**–**
	D/D or I/D	16 (17.78)	38 (40.0)	0.39 (0.18–0.82)	0.013	0.40 (0.14–1.12)	0.080	77.4%
A/C	I/I	13 (14.44)	15 (15.79)	0.79 (0.33–1.91)	0.611	1.62 (0.46–5.64)	0.450	3.8%
	D/D or I/D	24 (26.67)	8 (8.42)	2.76 (1.09–6.96)	0.032	3.80 (1.04–13.94)	0.044	65.1%
Other genotype combinations		66 (73.33)	87 (91.58)	1	–	1	**–**	**–**
A/C	D/D or I/D	24 (26.67)	8 (8.42)	3.95 (1.67–9.36)	0.002*	4.88 (1.38–18.01)	0.014*	91.1%

OR, Odd`s ratio; aOR, Adjusted Odd`s ratio; 95% CI, 95% confidence interval; RERI, relative excess risk due to interaction; AP, attributable proportion; S, synergy index;

^a^ Odd’s ratio and confidence interval was calculated by logistic regression.

^b^ Calculated by multivariate logistic regression and adjusted for age, gender, BMI, education level, occupational type, vaccination status and interval between vaccine doses, vaccine type of all doses, smoking status and alcohol abuse.

* Significant after Bonferroni correction.

## Discussion

Recent studies reported a number of evidence that rs4646994 or rs75603675 polymorphisms were individually found to be significant for susceptibility to COVID-19 and disease mortality. In present study, the findings were novel in highlighting a strong positive interaction between rs4646994 and rs75603675 polymorphisms of *ACE* and *TMPRSS2* genes, which strongly increase the risk of severe COVID-19 compared with mild COVID-19. However, this study had some limitations. Starting from July 2021, the SARS-CoV-2 Delta variant was actively spreading in Mongolia, with the outbreak continuing until December 2021 [19]. During the second half of 2021, the number of patients infected with the Delta variant who developed severe COVID-19 was relatively low, which was the main reason for the small sample size in our study. The small sample size may have reduced the statistical significance and led to a widening of the confidence intervals. Previous studies indicated that age (≥60 years), male gender, and some comorbidities are main risk factors for severe COVID-19 [20]. Regarding these risk factors, except arterial hypertension, were similar between groups in present study. This lack significant difference is may related to the study design, the sampling method and/or small sample size. On the other hand, all participants included in the study were fully vaccinated, which might be the reason of few cases of severe COVID-19 and these factors being balanced between the groups. This hypothesis is supported by the observation that in previous studies, the incidence of severe COVID-19 was decreased among vaccinated individuals, and the significance levels of other risk factors were modified after vaccination [21–23].

Recent studies have shown that rs75603675 of *TMPRSS2* is associated with the severity of COVID-19. In 2022, Villapalos-García G., et al has found that rs75603675 associated with severity of COVID-19 disease among the participants recruited from Spanish hospital [24]. Saba AA., et al explored the association between rs75603675 and COVID-19 among few sample size, which were collected from Bangladesh Institute. They reported in 2024, A allele was relatively higher among patients with severe COVID-19 [25]. In addition, Martínez-Gómez LE., has found rs75603675 A/A genotype frequency was significantly higher in deceased COVID-19 group [26]. Calcagnile M., et al mentioned hypotheses that rs75603675 may associated with increased accessibility of endocytic signal which led to the entry of SARS-CoV-2 into cells [27].

The rs4646994 is also well-studied polymorphism as the risk factor that contributes the pathogenesis of COVID-19 and it was approved with a number of evidence, the deletion of rs4646994 is strongly associated with increased risk of severe COVID-19 and its mortality [9,28–30]. The rs4646994 deletion upregulates ACE enzyme that lead to increased level of Ang II and ACE/ACE2 ratio [31]. Koka V. suggested that Ang-II down-regulates ACE2 expression via AT1 receptor mediated ERK/p38 MAP kinase signaling pathway [32]. Pagliaro P. mentioned that the higher level of Ang II and ACE/ACE2 ratio might explain worse outcomes in COVID-19 [33]. Similarly, Verdecchia P. and Banu N. reported ACE2 downregulation and increased level of ACE/ACE2 ratio aggravates inflammatory and thrombotic processes in COVID-19 disease [34,35].

Rossi ÁD., et al explored the relation between the expression levels of ACE2 and TMPRSS2 with COVID-19 [36]. They reported interesting data that indicates TMPRSS2/ACE2 expression level ratio was 3.29-fold higher in severe COVID-19 patients compared with mild COVID-19 patients. Also, Grisard HBdS., et al reported the expression level of ACE2 and TMPRSS2 are the potential risk factor for COVID-19 [37]. Previous reports clearly indicate the mechanism that the rs4646994 Del allele increases the activation of ACE, thereby increasing the production of Ang-II and decreasing the expression of ACE2, which leads to severe COVID-19. On the other hand, our findings by MDR analysis showed that the strong positive interaction between rs4646994 and rs75603675 polymorphisms and combination of A/C genotype of rs75603675 and the D/D or I/D genotype of rs4646994 is associated with 4.88-fold increased risk of severe COVID-19 compared with mild COVID-19. These data suggest an indirect relation between downregulation of ACE2 by the deletion allele of rs4646994 and TMPRSS2 may exists in the pathogenesis of severe COVID-19. The genetic interactions between ACE, ACE2, and TMPRSS2 should be considered in further studies.

## Conclusions

We found the strong positive-interaction between rs4646994 of *ACE* and rs75603675 of *TMPRSS2* that increases the risk of severe COVID-19 and it supports the pathogenesis of COVID-19 might be driven with SNP-SNP interaction of these genes.

## Supporting information

S1 Table Primer, probe sequences and restriction enzymes used for genotyping. PCR, Polymerase chain reaction; ARMS-PCR, Amplification refractory mutation system polymerase chain reaction; RFLP, Restriction fragment length analysis; qPCR, Real-time polymerase chain reaction; AT, Annealing temperature; RE, Restriction enzyme; Ref, reference.
(DOCX)

S2 Table Comparison of clinical manifestations between severe and mild COVID-19 groups. The values were given as numbers (proportion). P value was calculated by Chi-squared test.
(DOCX)

S3 Table Comparison of genotype frequencies of SNPs between groups (M vs A and S+M vs A). S, Severe COVID-19; M, Mild COVID-19; A, Asymptomatic; OR, Odd`s ratio; aOR, Adjusted Odd`s ratio; 95% CI, 95% confidence interval. ^a^Odd`s ratio and confidence interval was calculated by logistic regression. b Calculated by multivariate logistic regression and adjusted for age, gender, BMI, education level, occupational type, vaccination status and interval between vaccine doses, vaccine type of all doses, smoking status and alcohol abuse.
(DOCX)
