# Supplementary material for: The synergistic interaction between ACE and TMPRSS2 polymorphisms increases the risk of severe COVID-19
Source: PLoS One. 2026 Feb 24;21(2):e0343590. doi: 10.1371/journal.pone.0343590 (PMC12931805; doi:10.1371/journal.pone.0343590)
Supplement: S1 Table — PCR, Polymerase chain reaction; ARMS-PCR, Amplification refractory mutation system polymerase chain reaction; RFLP, Restriction fragment length analysis; qPCR, Real-time polymerase chain reaction; AT, Annealing temperature; RE, Restriction enzyme; Ref, reference. (DOCX) [file pone.0343590.s001.docx]

**S1 Table. Primer, probe sequences and restriction enzymes used for genotyping**

| **Gene** | **SNP** | **Method** | **Primers (5` to 3`)** | **AT** | **RE** | **Products size** | **Ref** |
| --- | --- | --- | --- | --- | --- | --- | --- |
| *ACE* | rs4646994 | PCR | CTG GAG ACC ACT CCC ATC CTT TCT | 60^O^C | - | I allele: 490 bp  D allele: 190 bp | (7) |
|  |  |  | GAT GTG GCC ATC ACA TTC GTC AGA T |  |  |  |  |
| *ACE2* | rs4240157 | ARMS-PCR | GCT GAG TTC TCA AAA TAA TGC CAT AGA T | 54^O^C | - | C allele: 194 bp  T allele: 243 bp | (7) |
|  |  |  | GAG GGT TGG TAA ATA GTG TTC AGT GG |  |  |  |  |
|  |  |  | GCC TCA GAA CAT TAC AGA ATC AAC CT |  |  |  |  |
|  |  |  | GCA TTT CTT TCC AAT CAT TAA GAG TTC A |  |  |  |  |
| *NR3C1* | rs56149945 | RFLP | CCA GTA ATG TAA CAC TGC CCC | 55^O^C | *Mlu*CI | C allele: 135, 92 bp  T allele: 135, 73, 19 bp | (12) |
|  |  |  | TTC GAC CAG GG AAG TTC AGA |  |  |  |  |
| *NR3C1* | rs41423247 | RFLP | GAG AAA TTC ACC CCT ACC AAC | 58^O^C | *Bcl* I | C allele: 418 bp  G allele: 263, 155 bp | (12) |
|  |  |  | AGA GCC CTA TTC TTC AAA CTG |  |  |  |  |
| *NR3C1* | rs10052957 | qPCR | GCA GAG GTG GAA ATG AAG GTG AT | 54^O^C | - | G allele: FAM  A allele: HEX | (13) |
|  |  |  | GGA GTG GGA CAT AAA GCT ATG ACA A |  |  |  |  |
|  |  |  | [FAM]ATT CAG ACT CAG TCA AGG[BHQ1] |  |  |  |  |
|  |  |  | [HEX]TAT TCA GAC TCA ATC AAG G[BHQ1] |  |  |  |  |
| *NR3C1* | rs6189/6190 | qPCR | TCC AAA GAA TCA TTA ACT CCT GGT AGA | 60^O^C | - | GG allele: HEX  AA allele: FAM | (13) |
|  |  |  | GCT CCT CCT CTT AGG GTT TTA TAG AAG |  |  |  |  |
|  |  |  | [FAM]ATC TCC CTT TTC CTG AGC A[BHQ1] |  |  |  |  |
|  |  |  | [HEX]ATC TCC CCT CTC CTG AG[BHQ1] |  |  |  |  |
| *TMPRSS2* | rs17854725 | ARMS-PCR | AGC ACG CTC TCG ACG CCC TCA | 64^O^C | - | G or A allele: 260 bp | (14) |
|  |  |  | AGC ACG CTC TCG ACG CCC TCG |  |  |  |  |
|  |  |  | CTT CCT GGG TCT GGG TGG GCT G |  |  |  |  |
| *TMPRSS2* | rs75603675 | RFLP | GCT CCT CAC ACC CGC TTT CG | 57^O^C | *Taq* I | A allele: 215, 18 bp | (14) |
|  |  |  | CCT GAG ATT AAA GCG AGA GCC AG |  |  | C allele: 233 bp |  |
| *TMPRSS2* | rs12329760 | RFLP | GCT GTC TGT TAC TGT CAC TCG GC | 55^O^C | *Msp* I | A allele: 159 bp | (14) |
|  |  |  | GGA CCA AAC TTC ATC CTT CCG |  |  | G allele: 138, 21 bp |  |
| *TMPRSS2* | rs4303795 | ARMS-PCR | CCT GAG TCC TTG CCC AGA GCC T | 64^O^C | *-* | G or A allele: 152 bp  Internal control: 299 bp | (14) |
|  |  |  | CCT GAG TCC TTG CCC AGA GCC C |  |  |  |  |
|  |  |  | GGC TCG AGT TTG GGT TAA GGA AG |  |  |  |  |
|  |  |  | GAG ACC TGG GAC CAT CCG GGG |  |  |  |  |

PCR, Polymerase chain reaction; ARMS-PCR, Amplification refractory mutation system polymerase chain reaction; RFLP, Restriction fragment length analysis; qPCR, Real-time polymerase chain reaction; AT, Annealing temperature; RE, Restriction enzyme; Ref, reference;
