# Supplementary material for: The synergistic interaction between ACE and TMPRSS2 polymorphisms increases the risk of severe COVID-19
Source: PLoS One. 2026 Feb 24;21(2):e0343590. doi: 10.1371/journal.pone.0343590 (PMC12931805; doi:10.1371/journal.pone.0343590)
Supplement: S2 Table — The values were given as numbers (proportion). P value was calculated by Chi-squared test. (DOCX) [file pone.0343590.s002.docx]

| **Characteristics** | **Severe COVID 19**  **N=90** | **Mild COVID 19**  **N=95** | **P value** |
| --- | --- | --- | --- |
| Intensive care internation | 0 (0.0) | 0 (0.0) | - |
| Supplemental oxygen (via nasal cannula) | 21 (18.9) | 12 (11.4) | 0.057 |
| Fever | 52 (57.8) | 37 (39.0) | 0.01 |
| Cough | 63 (70.0) | 56 (59.0) | 0.117 |
| Sore throat | 50 (55.6) | 46 (48.4) | 0.332 |
| Fatigue | 79 (87.8) | 71 (74.7) | 0.024 |
| Headache | 67 (74.4) | 48 (50.5) | 0.008 |
| Diarrhea | 12 (13.3) | 10 (10.5) | 0.556 |
| Dyspnea | 72 (80.0) | 41 (43.2) | <0.001 |
| Muscle pain | 35 (38.9) | 33 (34.7) | 0.558 |

**S2 Table. Comparison of clinical manifestations between severe and mild COVID-19 groups.**

The values were given as numbers (proportion)

P value was calculated by Chi-squared test.
