# Supplementary material for: The synergistic interaction between ACE and TMPRSS2 polymorphisms increases the risk of severe COVID-19
Source: PLoS One. 2026 Feb 24;21(2):e0343590. doi: 10.1371/journal.pone.0343590 (PMC12931805; doi:10.1371/journal.pone.0343590)
Supplement: S3 Table — S, Severe COVID-19; M, Mild COVID-19; A, Asymptomatic; OR, Odd`s ratio; aOR, Adjusted Odd`s ratio; 95% CI, 95% confidence interval. aOdd`s ratio and confidence interval was calculated by logistic regression. b Calculated by multivariate logistic regression and adjusted for age, gender, BMI, education level, occupational type, vaccination status and interval between vaccine doses, vaccine type of all doses, smoking status and alcohol abuse. (DOCX) [file pone.0343590.s003.docx]

**S3 Table. Comparison of genotype frequencies of SNPs between groups (M vs A and S+M vs A)**

| **refSNP ID** | **Model** | **Genotype** | **Frequency** | | | **M vs A** | | **S + M vs A** | |
| --- | --- | --- | --- | --- | --- | --- | --- | --- | --- |
|  |  |  | **S**  **N=90** | **M**  **N=95** | **A**  **N=90** | **OR (95% CI)^a^** | **aOR (95% CI)^b^** | **OR (95% CI)^a^** | **aOR (95% CI)^b^** |
| rs4646994 | Dominant | D/D+I/D | 0.556 | 0.516 | 0.444 | 1.33 (0.75-2.37) | 1.64 (0.71-3.79) | 1.44 (0.87-2.39) | 1.79 (0.89-3.58) |
| rs4240157 | Recessive | C/C | 0.078 | 0.032 | 0.067 | 0.46 (0.11-1.88) | 0.44 (0.07-2.97) | 0.80 (0.28-2.27) | 0.69 (0.17-2.82) |
| rs41423247 | Recessive | C/C | 0.122 | 0.095 | 0.078 | 1.24 (0.44-3.48) | 1.66 (0.35-7.78) | 1.44 (0.58-3.54) | 1.47 (0.37-5.76) |
| rs56149945 | Overdominant | A/G | 0.033 | 0.011 | 0.022 | 0.46 (0.04-5.25) | 1.19 (0.05-27.4) | 0.97 (0.17-5.41) | 1.30 (0.1-16.93) |
| rs10052957 | Recessive | A/A | 0.033 | 0.021 | 0.011 | 1.91 (0.17-21.48) | 2.0 (0.09-42.13) | 2.47 (0.28-21.48) | 1.44 (0.12-17.4) |
| rs6189/6190 | Recessive | Non GG | 0.022 | 0.021 | 0.011 | 1.91 (0.17-21.48) | 0.61 (0.02-16.2) | 1.97 (0.22-17.86) | 0.75 (0.06-9.45) |
| rs12329760 | Recessive | A/A | 0.267 | 0.200 | 0.211 | 0.93 (0.46-1.91) | 0.65 (0.23-1.78) | 1.06 (0.58-1.94) | 0.89 (0.38-2.09) |
| rs4303795 | Overdominant | A/G | 0.278 | 0.179 | 0.189 | 0.94 (0.44-1.97) | 1.06 (0.47-2.39) | 1.26 (0.67-2.37) | 1.34 (0.68-2.61) |
| rs75603675 | Overdominant | A/C | 0.411 | 0.242 | 0.367 | 0.55 (0.29-1.04) | 0.62 (0.31-1.23) | 0.83 (0.49-1.41) | 0.92 (0.53-1.62) |
| rs17854725 | Dominant | A/A | 0.756 | 0.695 | 0.667 | 1.14 (0.61-2.11) | 0.84 (0.34-2.08) | 1.31 (0.76-2.26) | 0.99 (0.47-2.12) |

S, Severe COVID-19; M, Mild COVID-19; A, Asymptomatic; OR, Odd`s ratio; aOR, Adjusted Odd`s ratio; 95% CI, 95% confidence interval.

^a^Odd`s ratio and confidence interval was calculated by logistic regression.

^b^Calculated by multivariate logistic regression and adjusted for age, gender, BMI, education level, occupational type, vaccination status and interval between vaccine doses, vaccine type of all doses, smoking status and alcohol abuse.
